# Supplementary material for: The retroelement-derived human protein PEG10 is a regulator of mRNA splicing in neurons
Source: bioRxiv. 2026 May 24:2026.05.21.727000. Preprint. [Version 1] doi: 10.64898/2026.05.21.727000 (PMC13228445; doi:10.64898/2026.05.21.727000)
Supplement: Supplement 3 [file NIHPP2026.05.21.727000v1-supplement-3.pdf]

bioRxiv preprint doi: https://doi.org/10.64898/2026.05.21.727000; this version posted May 24, 2026. The copyright holder for this preprint (which was not certified by peer review) is the author/funder, who has granted bioRxiv a license to display the preprint in perpetuity. It is made available under a CC-BY 4.0 International license.

Supplemental Figures

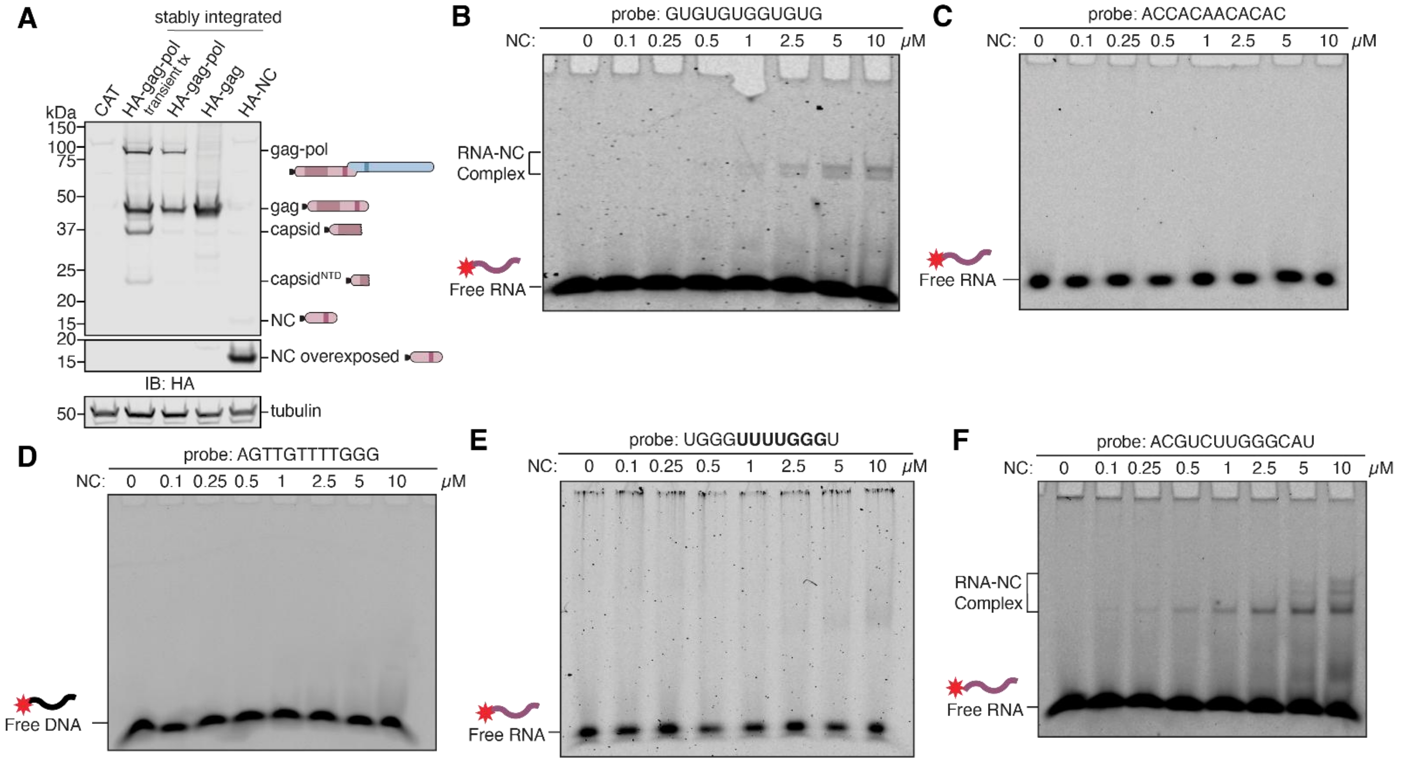

Figure S1. Stable cell line generation and additional fEMSAs showing NC-specific RNA-binding preference, related to Figure 1D-F.(A) Western blot of whole cell lysate from Flp-In-293 cells stably expressing control chloramphenicol acetyltransferase (CAT), HA-gag-pol, HA-gag, or HA-NC. In lane 2, whole cell lysate of Flp-In-293 cells transiently transfected with HA-gag-pol for 24 hours was performed to compare PEG10 expression and self-processing levels before and after stable integration. Lysate was probed by western blot for HA and tubulin. HA-tagged NC runs at about 15 kDa and was overexposed for ease of visualization at bottom. **(B)** fEMSA of PEG10 NC incubated with 0.5 nM GUGUGUGGUGUG RNA oligo with a 5' Cy5.5 probe. (n=3). **(C)** fEMSA of PEG10 NC incubated with 0.5 nM ACCACAACACAC RNA oligo with a 5' Cy5.5 probe. (n=3). **(D)** fEMSA of PEG10 NC incubated with 0.5 nM AGTTGTTTTGGG DNA oligo with a 5' Cy5.5 probe. (n=2). **(E)** fEMSA of PEG10 NC incubated with 0.5 nM UGGGGUUUUGGGU RNA oligo with a 5' Cy5.5 probe. (n=2). **(F)** fEMSA of PEG10 NC incubated with 0.5 nM ACGUCUUGGGCAU RNA oligo with a 5' Cy5.5 probe. (n=2).

bioRxiv preprint doi: https://doi.org/10.64898/2026.05.21.727000; this version posted May 24, 2026. The copyright holder for this preprint (which was not certified by peer review) is the author/funder, who has granted bioRxiv a license to display the preprint in perpetuity. It is made available under a CC-BY 4.0 International license.

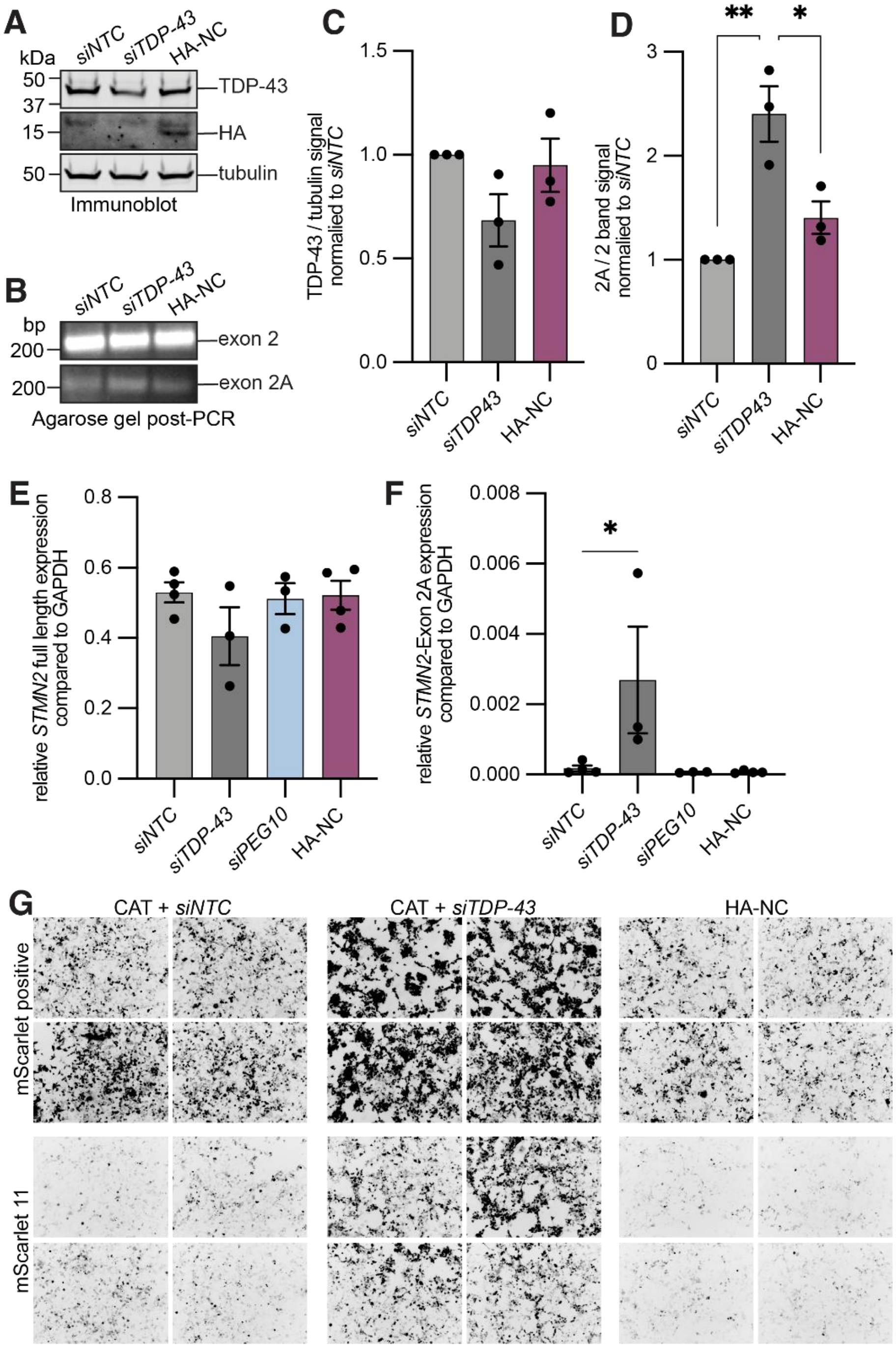

Figure S2. NC does not regulate splicing of *STMN2* in the same manner as TDP-43, related to Figures 2 and 4. (A) Representative western blot of SH-SY5Y cells transfected with *siNTC, siTDP-43*, or HA-NC. Cells were harvested 48 hours post transfection and probed for HA, TDP-43, and tubulin. (n=3). **(B)** Representative PCR results of SH-SY5Y cells transfected as in (A) for *STMN2* exon 2A or 2 visualized by 2% agarose gel stained with SYBR Gold (n=3). **(C)** Quantification of western blot results from (A). (n=4). There are no significant differences in TDP43 levels. **(D)** Quantification of agarose gel comparing the presence of *STMN2* exon 2A to exon 2. For (C-D), a one-way ANOVA was performed and compared to the means of each other. **(E)** qPCR for full-length *STMN2,* **(F)** qPCR for *STMN2* exon 2A. For (E-F), a one-way ANOVA was performed and compared to the mean of si*NTC*. (n=3 or 4). **(G)** Representative images of control CAT or HA-NC stable cell lines transfected with mScarlet reporters of TDP-43-dependent cryptic exon inclusion. mScarlet positive control, and reporter #11 were selected for testing. CAT HEK293 cells were transfected with either *siNTC* or *siTDP-43* for 24 hours, then all cell lines were transfected with mScarlet splice reporter constructs for an additional 24 hours. Images were taken at 20X magnification on an EVOS microscope, with 4 images per condition to show representative fields of view (n=3). *p<0.05, **p<0.01, ***p<0.001, and ****p<0.0001.

bioRxiv preprint doi: https://doi.org/10.64898/2026.05.21.727000; this version posted May 24, 2026. The copyright holder for this preprint (which was not certified by peer review) is the author/funder, who has granted bioRxiv a license to display the preprint in perpetuity. It is made available under a CC-BY 4.0 International license.

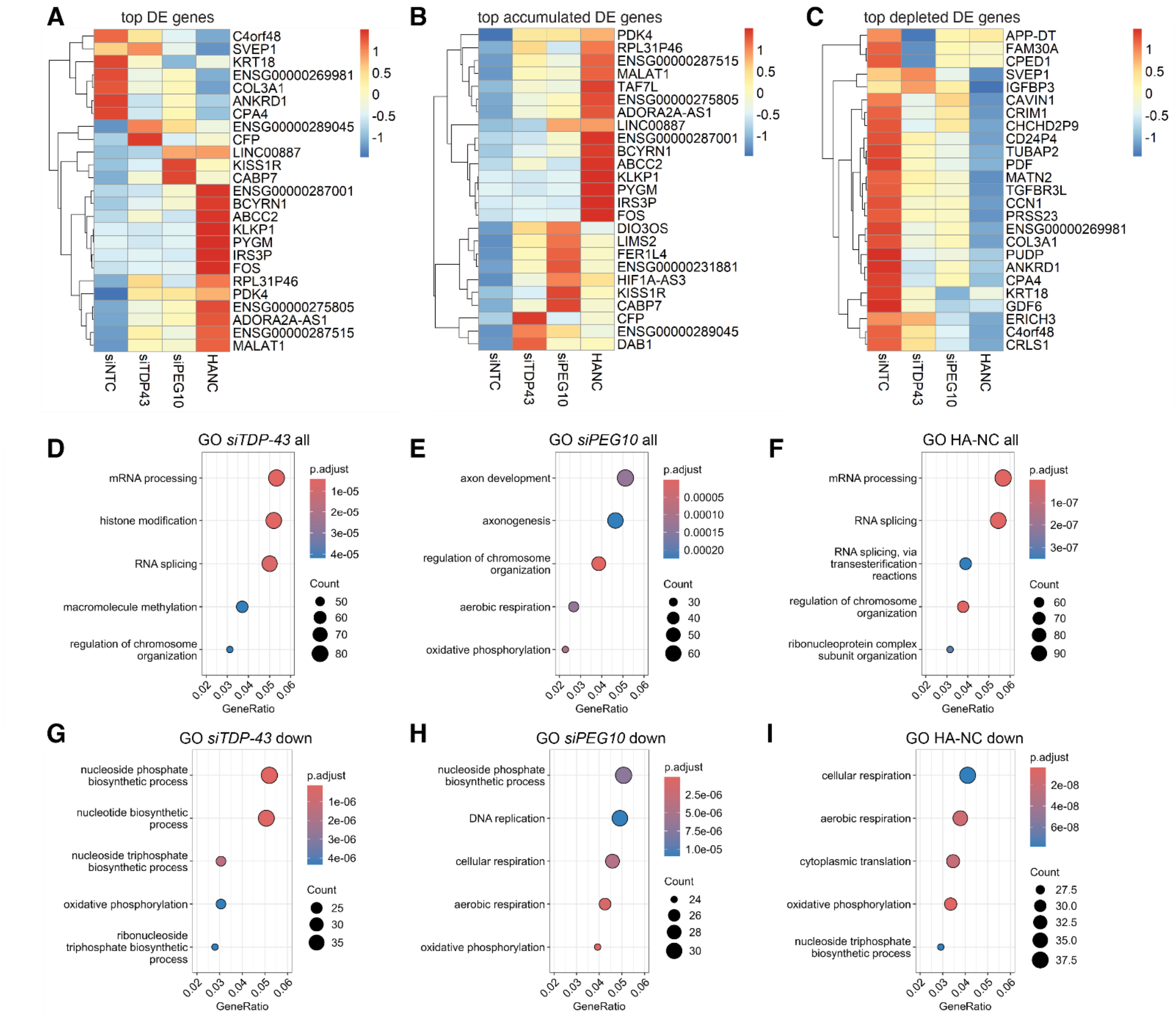

Figure S3. NC leads to widespread changes to gene regulation pathways, related to Figure 3.(A-C) Heatmap of **(A)** the most differentially expressed (DE) genes across all conditions **(B)** most accumulated DE genes across all conditions and **(C)** most depleted DE genes across all conditions. (n=4). For each heatmap, the top genes across all conditions relative to *siNTC* were combined. Genes were then ranked according to $\log_2$FoldChange across the combined dataset. Genes were then color coded by row Z-score. **(D-F)** Top changed pathways of gene expression changes in either direction in cells transfected with **(D)** *siTDP-43,* **(E)** *siPEG10*, or **(F)** HA-NC by GO-term enrichment analysis. **(G-I)** Top downregulated pathways of gene expression changes in cells transfected with **(G)** *siTDP-43,* **(H)** *siPEG10*, or **(I)** HA-NC by GO-term enrichment analysis. The top five GO-terms using a $\log_2$FoldChange cutoff of 0.1 were ranked by adjusted p-value. Adjusted p-value is shown by color, and the size of the datapoint reflects the number of genes enriched in the pathway.

bioRxiv preprint doi: https://doi.org/10.64898/2026.05.21.727000; this version posted May 24, 2026. The copyright holder for this preprint (which was not certified by peer review) is the author/funder, who has granted bioRxiv a license to display the preprint in perpetuity. It is made available under a CC-BY 4.0 International license.

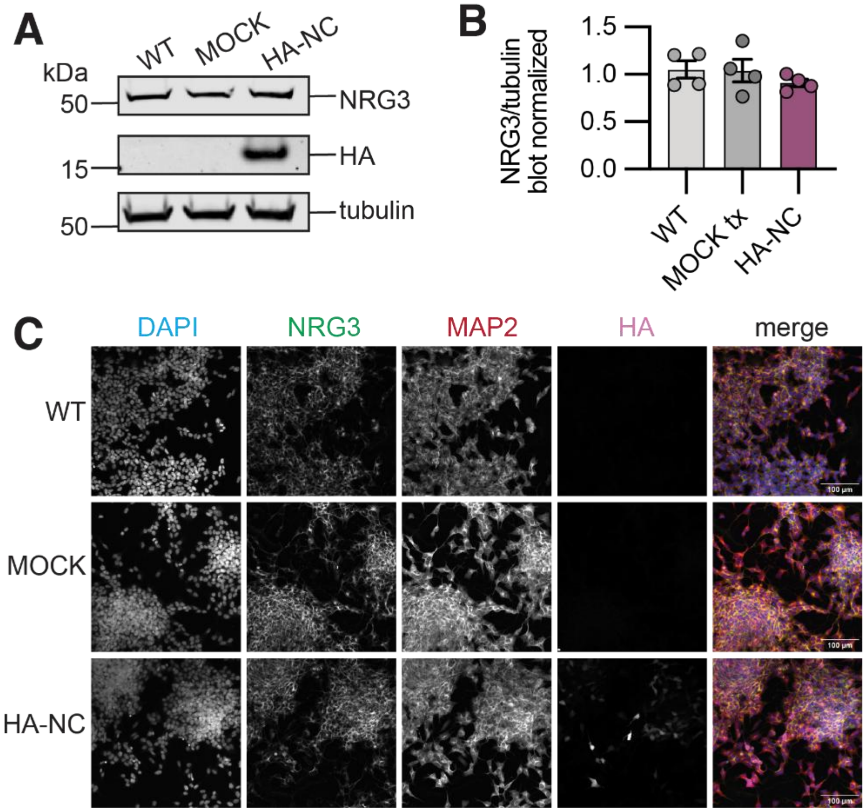

Figure S4. NRG3 abundance is not altered in bulk lysate of NC-expressing SH-SY5Y cells, related to Figure 5. (A) Representative western blot of WT SH-SY5Y cells compared to cells that have been mock transfected with lipofectamine alone (MOCK), or transfected with HA-NC.Cells were harvested 48 hours post transfection and probed for HA, NRG3, and tubulin. (n=4). **(B)** Quantification of western blot results from A. (n=4). There are no significant differences in NRG3 levels by one-way ANOVA. **(C)** Representative images from IF staining of WT SH-SY5Y cells compared to MOCK or HA-NC transfected cells. Cells were stained with NRG3, MAP2, and HA and coverslips were mounted with Prolong Gold with DAPI. Z-stack images were taken on a Nikon AXR at a 2048 size at Nyquist. Scale bar = 10 μm, and contrast has been increased for ease of visualization. (n=3).

bioRxiv preprint doi: https://doi.org/10.64898/2026.05.21.727000; this version posted May 24, 2026. The copyright holder for this preprint (which was not certified by peer review) is the author/funder, who has granted bioRxiv a license to display the preprint in perpetuity. It is made available under a CC-BY 4.0 International license.

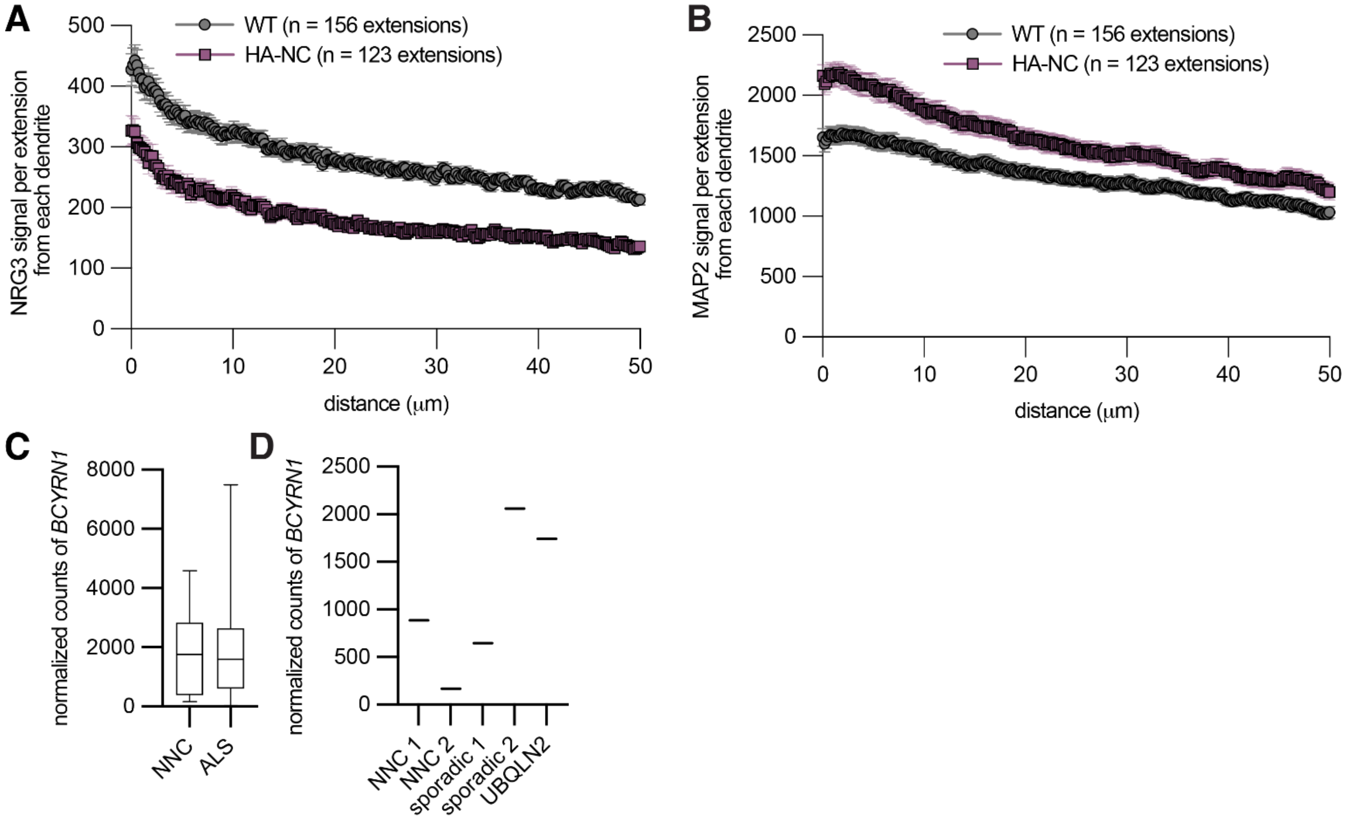

Figure S5. MAP2 is not depleted in iNeurons expressing NC and BCYRN1 is altered in human ALS, related to Figures 6 and 7.

(A-B) mean **(A)** NRG3 or **(B)** MAP2 signal from dendrites starting at the branch point from the soma in differentiated iNeurons of either WT or HA-NC expression (n=1 quantified experiment). The number of extensions is shown in each legend. (C) Normalized counts of *BCYRN1* for NNC and sporadic ALS samples for Target ALS patient samples analyzed in Figure 7D-F. Counts were not significantly different by Student's T test. (D) Normalized counts of *BCYRN1* for targetALS patient samples ananlyzed in Figure 7H, illustrating a similar expression profile with the exclusion of exon 9 in *UBQLN2*-mediated fALS and one sALS case. Datapoints are shown as lines to highlight that only one sample is evaluated per column.

bioRxiv preprint doi: https://doi.org/10.64898/2026.05.21.727000; this version posted May 24, 2026. The copyright holder for this preprint (which was not certified by peer review) is the author/funder, who has granted bioRxiv a license to display the preprint in perpetuity. It is made available under a CC-BY 4.0 International license.

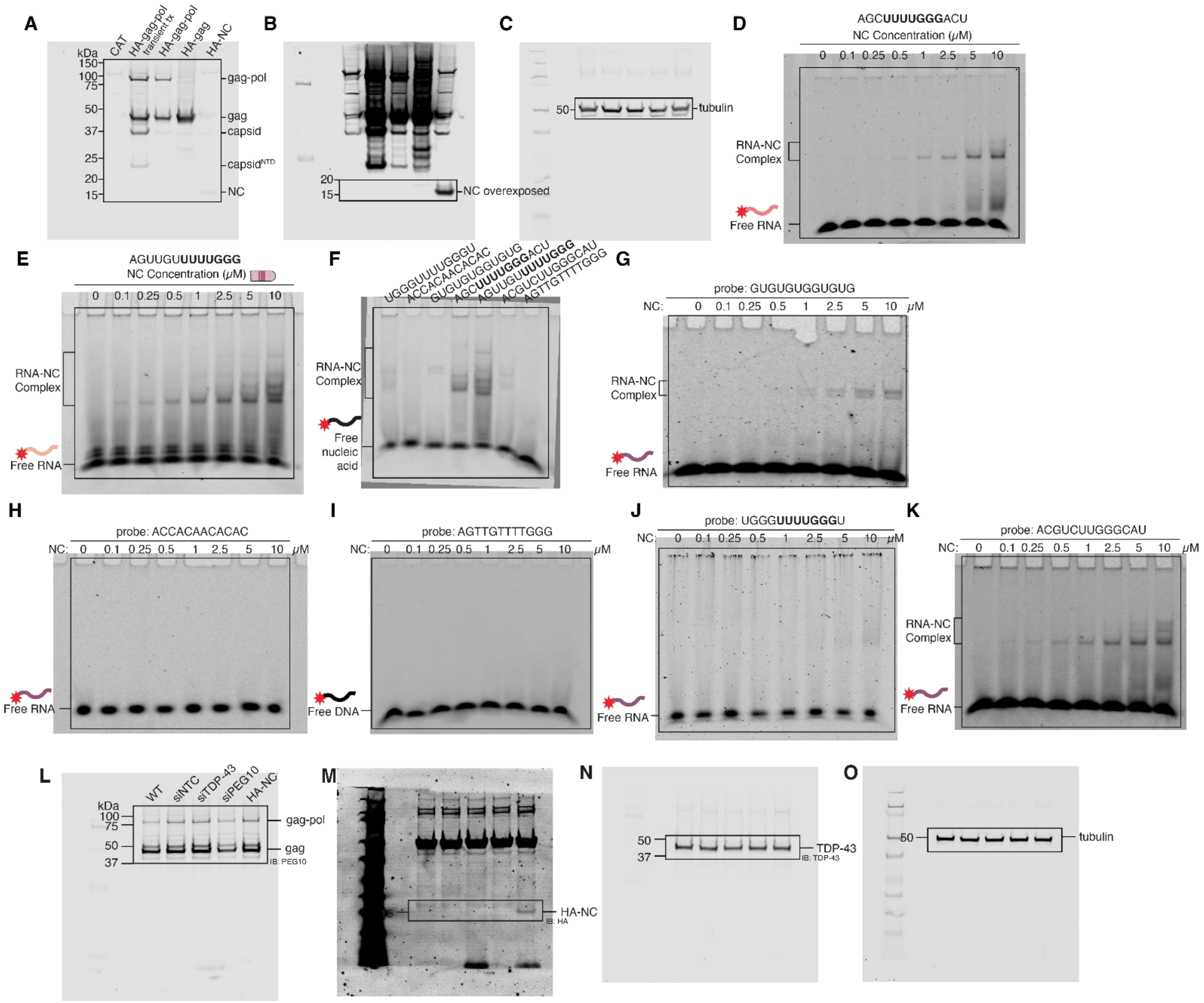

Figure S6. Uncropped western blots and fEMSAs, related to Figures 1 and 2.
(A-C) Uncropped western blot from Figure S1A, **(D-K)** Uncropped fEMSAs from Figure 1E-G and Figure S1B-F, **(L-O)** Uncropped western blots from Figure 2A.

bioRxiv preprint doi: https://doi.org/10.64898/2026.05.21.727000; this version posted May 24, 2026. The copyright holder for this preprint (which was not certified by peer review) is the author/funder, who has granted bioRxiv a license to display the preprint in perpetuity. It is made available under a CC-BY 4.0 International license.

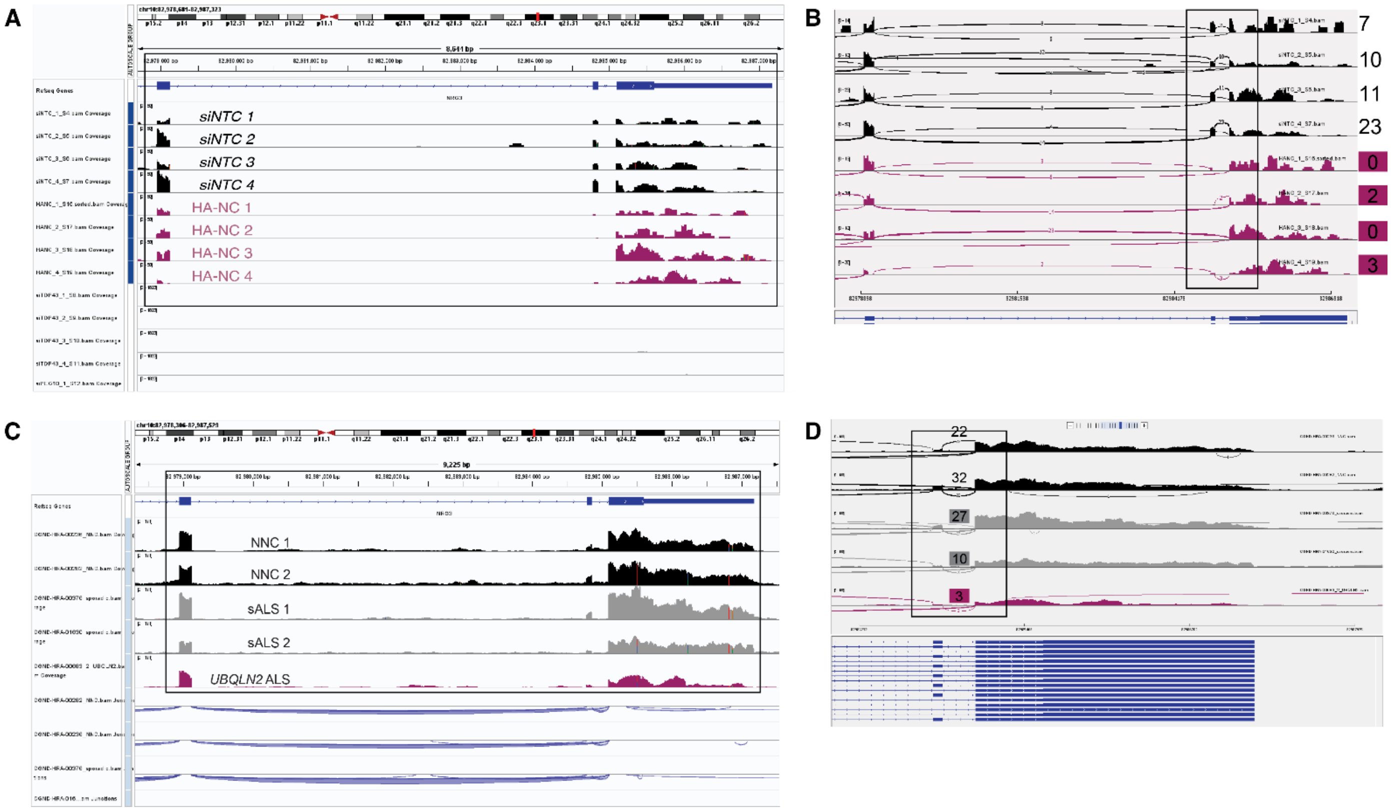

Figure S7. Uncropped IGV snapshots, related to Figures 4 and 7. (A-B) uncropped IGV snapshots from Figure 4F-G. **(C-D)** Uncropped IGV snapshots from Figure 7F.

Table S1: Gene expression changes and pathway analysis of SH-SY5Y RNA-Seq data. (A-C) Differentially expressed genes using a log_2FoldChange cutoff of 0.1 and p adjusted value cutoff of 0.05 for all three relevant comparisons. **(D-F)** GO-term enrichments for NC-expressing SH-SY5Y cells compared to *siNTC*-expressing cells including upregulated genes, all significantly changed genes, or downregulated genes with a log_2FoldChange cutoff of 0.1 and p adjusted value cutoff of 0.05. **(G-I)** GO-term enrichments for *siPEG10*-expressing SH-SY5Y cells compared to *siNTC*-expressing cells including upregulated genes, all significantly changed genes, or downregulated genes with a log2fold cutoff of 0.1 and p adjusted value cutoff of 0.05. **(J-L)** GO-term enrichments for *siTDP-43*-expressing SH-SY5Y cells compared to *siNTC*-expressing cells including upregulated genes, all significantly changed genes, or downregulated genes with a log_2FoldChange cutoff of 0.1 and p adjusted value cutoff of 0.05.

Table S2: Top splice changes between TDP-43 depletion, PEG10 depletion, and NC overexpression. Top local splice variants (LSVs) for *siTDP-43*, *siPEG10*, and HA-NC transfected cells compared to siNTC using MAJIQ Splice changes were determined using a deltapsi cutoff of 0.1 and a confidence interval cutoff of 0.9 and all LSVs were compiled for each condition.
